# Supplementary material for: Analysis of the AIRE Gene Promoter in Patients Affected by Autoimmune Polyendocrine Syndromes
Source: Int J Mol Sci. 2024 Feb 24;25(5):2656. doi: 10.3390/ijms25052656 (PMC10932416; doi:10.3390/ijms25052656)
Supplement: Supplementary file 1 [file ijms-25-02656-s001.zip › ijms-2860675-supplementary.pdf]

Supplementary Material

Analysis of the *AIRE* Gene Promoter in Patients Affected by Autoimmune Polyendocrine Syndromes

Annamaria Cudini [1,†], Caterina Nardella [1,†], Emanuele Bellacchio [2], Alessia Palma [1], Domenico Vittorio Delfino [3], Corrado Betterle [4], Marco Cappa [5] and Alessandra Fierabracci [1,*]

[1] Bambino Gesù Children's Hospital, IRCCS, 00165 Rome, Italy; annamaria.cudini@opbg.net (A.C.); caterina.nardella@opbg.net (C.N.); alessia.palma@opbg.net (A.P.)
[2] Molecular Genetics and Functional Genomics, Bambino Gesù Children's Hospital, IRCCS, 00165 Rome, Italy; emanuele.bellacchio@opbg.net
[3] Section of Pharmacology, Department of Medicine and Surgery, University of Perugia, 06129 Perugia, Italy; domenico.delfino@unipg.it
[4] Padua University, 35128 Padua, Italy; corrado.betterle@unipd.it
[5] Research Unit for Innovative Therapies in Endocrinopathies, Bambino Gesù Children's Hospital, IRCCS, 00165 Rome, Italy; marco.cappa@opbg.net
* Correspondence: alessandra.fierabracci@opbg.net
† These authors contributed equally to this work.

1. Supplementary Figures and Tables

1.1. Supplementary Figures

```
H.sapiens      ggcca-----------cccccctccaggccatgcctgcggggccaccacagcctcagcatcattgcaggccccaggcctc
P.troglodytes  ggcca-----------cccccctccaggccatgcctgcggggccaccacagcctcagcatcattgcaggccccaggtctc
M.mulatta      ggcca------------tcctactccaggccatgcctgcgcggctgccacagcctcggcaccattgcaggccccggcctc
U.arctos       gggcaggtgtctcctctgtggcccttgtccctctgccccaggcca-cgatgggccactat------------gggtcccc
B.taurus       tgtctcatgtccctgccccccacccagaccatgctgcctgggcca-catgggctcagggtccatggacaccctggtcctc
S.carolinensis ggttgagcactctc------------------------------------------------------aggccaggctcc

H.sapiens      tgcacctggtcttgttttactgggggcactgtccccactcacgtccacctgggaccctcggctcctgtccactgcagctc
P.troglodytes  tgcacctggtcttgttttactgggggcattgtccccactcacgtccacctgggaccctcggctcctgtccactgcagctc
M.mulatta      tgcacctgctcctgctttactgggggcactgtccccacccatgtccacctgggacctttggctcctgcccactgtagctc
U.arctos       tgcactcgcccaccatagcctgggatgcggagccc--------------------------------ccctctgctgcaa
B.taurus       tgcccctgcccacccattgctgggtatgctgttcccatctcatg-----------------gaccaggacccctgctgtga
S.carolinensis cgcacctgctcccatacccctgcaagcctctgccccctc----------------------------tccacagcagctg

H.sapiens      ccctcccgga-caccttcccagatgcccccggaagctc-ctgtccaggccacagcatccctcagcctctgtcactggtcc
P.troglodytes  ccctccagga-caccttcccagatgcccccggaagctc-ctgtccaggccacagcatccctcagcctctgtcactggtcc
M.mulatta      ccctccagga-caccttcctagatgcccctggaagttc-ctgtccaggccacagcatccctcagcctctgtcactggtcc
U.arctos       ctcccctgggccggcttcc-----------------------------ccacatcatcctccagcctctgtcaccagccc
B.taurus       ttctccaggg-agccttct-----------------------------tcacttcccccagaagcttctgtccccaaccc
S.carolinensis ccctccagga-agccttccgccattcctctgaggctcctccctccaagctacgtgatccctcagtctctgttaccagtcc

H.sapiens      taggaagaccctttgggagctctcactca----------------------------------gggcc-acactcaggac
P.troglodytes  taggaagaccctttgggagctctcactcc----------------------------------gggcc-acactcaggac
M.mulatta      taggaagatccctagggagccctccctca----------------------------------gggcc-acactcagggg
U.arctos       taggaaggctctgggggcgccccccccccccggggctgtgag---------caggggctcctggggcc-atactcaggtg
B.taurus       taggaagacccctggggtgcccctccctc----ggctgtgagccagccagacaggggctcccatggctaatgctcagata
S.carolinensis caagaaggctccttgggggaccccaccca----ggcaa----------ggacagggactcccacggcc-------agggg

H.sapiens      c--cccgttgtggggctggccgccttccttcta--------------aagcacctggaggaaggaaggaggggtcaatgc
P.troglodytes  c--cccgttgtggggctggccgccttccttcta--------------aagcacctggaggaaggaaggaggggtcaatgc
M.mulatta      c--cctgttgtggggccggccatcttccttcta--------------aagcacctggaggaaggaagtaggggtcaatgc
U.arctos       catcagtcc-cagggtcagctgccttctccgag-------------------ctgg----aggaaagggagggtagattg
B.taurus       tgtctgtccatggggttagtcaccttcctagaagctttacttcccaacacacccag----aggaggtagaggatggattg
S.carolinensis catttgtccgtggggccagctatcttcttgaagcttccttttct---------------------------agtgtacct

H.sapiens      gagcc-tcaatccccaggcgagtgtgccccttttaaagatgagggaaccgaggctcagagaaggaaag-gacttgcctgg
P.troglodytes  gagcc-tcaatccccaggcgagtgtgccccttttaaagatgagggaaccgaggctcagagaaggaaag-gacttgcctgg
M.mulatta      gagcc-tcggtctccaggcgaatgcgccctttttaaagatcagggaactgaggctcagagaaggaaag-ggcttgcc-gg
U.arctos       cggct-gggtcccccaggggggatgtgctcatgtgaaagatgagggggttgagtctctgagagggaaagcgactggcccag
B.taurus       accctggggactcccagtggaaagtgtccatttcaaagatga---------------gagaaggagagacacttgtcccg
S.carolinensis ggact-gggcaccccagctaaatgtgctaaggttaaagctgg---gactgaggctcagagaaggaatg-gacgtgcccag

                                                                                rs117557896
                                                                                |
H.sapiens      cgtcacacagctagcctaa-gatggtgaggtcagga-gctccctggcaacaccca-ggc--tgccgtaccgtctcctgca
P.troglodytes  cgtcacacagccagcctaa-gatggtggggtcaggaggctccccggcaacgccca-ggc--tgccgtaccgtctcctcca
M.mulatta      catcacacagccagcctga-gacggtgaagtcagcagcctccccggggacgcccagggc--tgcggtaccgtctcctcca
U.arctos       catcacacagcttccaggc-aatgc-----cccgtgctgtg-ccacgggcacac---------------actgtcctcca
B.taurus       catcacccagactccaggt-gatgc-----ccagcactgaacccactgatacactaagtcataacccagggcaccctta
S.carolinensis agtcacacgacagggaggtcgaagc-----tcaaaagcccaccaccagcaccctgggt------gtccccactcctcca

H.sapiens      tcagactgag--cctccatcgggctcctcccacagccccaggc--gggcccctgagtaggggctctcagct-tgtgtgga
P.troglodytes  tcagactgag--cctccatccggctcctcccacagccccaggc--gggcccctgagtaggggctctcagct-tgtgtgga
M.mulatta      tcagactggg--cctccacccggctcctcccacagccccaggc--gggcccctgagtaggggctttcagct-tgtgtgga
U.arctos       tcagacaggcactcctcccttggacccttctgcag-cccaggcccagggccctgcgcactcccca---------------
B.taurus       tcagaca-gcacccctcactggacccctcctacagccccagactagggattctgagcagcagctg---------------
S.carolinensis tcagaccgggaccctccatctgactcctccagc---cctagact-gggacgttgagcagagaccctgagccaagtgtgga

H.sapiens      ggtcccccaggaacaccaccccgatccagc-----ctccatggaggctcttgccggccactgggaggggccggtgcaccc
P.troglodytes  ggtcccccaggaacaccaccccgatccagc-----ctccatggaggctcttgccggccactgggaggggccagtgcaccc
M.mulatta      ggttccccagggacaccaccccgatccagc-----ctccatggaggctcttgccggccactgggaggggccattgcaccc
U.arctos       ------ccagggaccctgccccaatcggcc----------cgacggtccctgcctgagtgccggagcccctgggcagccc
B.taurus       ------cggaggcagtagtgcc---------------------cactccctgccccggctc---agctccctggaggctt
S.carolinensis cgtcccccagggtcagggctcc-atctagcagcaaccccgtggacgtctctatcagagccacgtggtggtgctgcaggtg
```

```
                                                          rs547103905
                                                          |
H.sapiens      tgggca------gccctgccagggccctgagacccgagcctccccgccgaggcacctgtctcggcttgccccattcg
P.troglodytes  tgggct------gccctgccagggccctgagacccgagcctccccgccgagggcacctgtctcggcttgccccattcg
M.mulatta      caggca------gccctgccaggccct-----------------------gaggcctgtcccggcttgccccattcg
U.arctos       cgtggg------cctcaggggaggtcaa----------------------gggcacctgcgttggctgttgcccatctg
B.taurus       catgcgctggccctggcagctgctgga-----------------------ggacacccagcactgcttcccccatttg
S.carolinensis tggccg------gcccagaggaggtctg----------------------gggcac-tgtcctggctt---ctcaccag

                                                            rs371261300
                                       -402S (C/G)          |  Ets-C
                                       |                    ▼▼▼▼▼▼▼▼
H.sapiens      agcagggccctc----------gccgaggcaggacagggccacattcggaagtgagagttctctgagtcccgcacagagc
P.troglodytes  agcagggccctc----------gccgaggcaggacagggccacattcggaattgagagttctctgagtcccgcacagagc
M.mulatta      agcagggccctg----------gggagggcaggacggggcacactgggaagtgagaggcctctgagtcccgcgcagagc
U.arctos       aacagaaccacagttcaggaattgagaggccccccctagccccctcacaga----gggtcggccctggtcc-------
B.taurus       aacagtgccccg----------tgggatgctggccggaaccacgttttgggaattgagtttggacgcttctttacaacgg
S.carolinensis gacagga---------gggaagccggagcccacccctgaacccccttctgt--------------------------gg

                                                       AP1                Ets-B
                                                   ▼▼▼▼▼▼▼▼▼▼▼       ▼▼▼▼▼▼▼▼
H.sapiens      gagtctctgtccccagccccccaaggcagctgccc----tggtgggtgagtcaggccaggcccggagacttcccgagagcg
P.troglodytes  gagtctctgtccccagccccccaaggcagctgccc----tggtgggtgagtcaggccaggcccggagacttcccgagagcg
M.mulatta      aagtctccgtccccagccccccaaagcagctgccc----tggtgggtgagtctggcc-ggtgcggagacttcccaggagcg
U.arctos       -agaagcttcaacagcctcctggcagg----------tgtggaagtggggtggct-ggggccttgtctttactggatta
B.taurus       aaagaaaagtcaacagcctgccga-agg----------tgttcaagggcgctggca-ggtggctgggaccttggggaact
S.carolinensis gtcggtgggccagagccaccagaggtgggtcccagaggcggggagcaggctggct-gcccaggcaccttctcttgatca

                                                    rs751032    rs184978263           rs1048356976
                 rs934375604         Ets-A          |  WT1      |CCAAT BOX   GC BOX   |
                 |                  ▼▼▼▼▼▼▼         |▼▼▼▼▼▼▼▼▼  |▼▼▼▼▼▼▼▼▼▼▼ ▼▼▼▼▼▼▼▼▼▼▼
H.sapiens      agggagggacagcagcgcctccatcacagggaagtgtccctgcgggaggccctggccctgattgggcgccggggcggagc
P.troglodytes  agggagggacagcagcgcctccatcacagggaagtgtccccgcgggaggccctggccctgattgggcgccggggcggagc
M.mulatta      agggagggacgcagcgcctccgtcacagggaagcgtccccgcgggaggctctggccctgattgggcgccggggcggagc
U.arctos       cagggacgattccatccctgcccc--------------------tccctccggccctgattggct-ctggggaagcac
B.taurus       cagtcacggggaagtttctggcccctgaggaa-------------gctgcctgggccctgattggc---------ggagc
S.carolinensis caggg--------------------------------cactttgcagtcctcgcctgattggctcctggg--ccggc

                                     TATA box
               ▼                    ▼▼▼▼▼▼▼▼▼▼▼
H.sapiens      ggcctttgctctttg----cgtggtcgcgggggtataacagcg------------------------------------
P.troglodytes  ggcctttgctctttg----cgtggtcgcgggggtataacagcg------------------------------------
M.mulatta      ggcctttgctctttg----cgtggtcgcgggggtataacagcg------------------------------------
U.arctos       agcctttgctctttcggtgggggggagtcggctataatagag---actgggcactggctgggctc-----agtccgtgg
B.taurus       ggccctcgctctctc----ccaggagacgcaggtataacagac---tccggccccgaggaggccgg-----ag-----ga
S.carolinensis agcctttgccctttc----ctgggtgg-gccagtataacagcaggcccctggcccatgcagggctctccagagctggcaa

H.sapiens      --------------------------------------gcgcgcgtggctcgcagac------------------------
P.troglodytes  --------------------------------------gcgcgcgtggctcgcagac------------------------
M.mulatta      --------------------------------------gcgcgcgtggctcgcagacccagggagacgggcgga-------
U.arctos       aggtgcccgcccaggtgcgccggccaggtgtgccccgcccaggtgcccggcaggcaggggctgcgcaaggcca----
B.taurus       agctgtcaggactccctctccccgagcaggtgagcccgcccaggtgtcc-ccggcgcaggcggagctcaaggacca----
S.carolinensis gagtcaattctcccagctgcctggcacctggtgcctggcgcacagggttctggcccgggagcacgggcggggcaggca

                   rs868650327
                   |
H.sapiens      ---cggggagacgggcgggcgcacagccggcgcggaggcccccacagcccgccggacccgaggccaagcgagggcgctgc
P.troglodytes  ---cggggagacgggcgggcgcacagccggcgcggaggcccccacagcccgccaggacccgaggccaagcgagggcgctgc
M.mulatta      ---cgggcgggcgggcgggcgcacagccggcgcggacgcccccacagcccgcccggacccgaggccaggcgagggcgctgc
U.arctos       ----------------ggaggcagagatggccagacgctccacaagtccagtggcacccaaggccgcgggacccagg
B.taurus       ----------------ggaggcagacacggcccggacacccccacaaggcagcggcgccctgaggccccaagacccagg
S.carolinensis gaccgctctgacagccagg---------agctcagagggactccaaggacga-gggaccctgaggcggagggacgccgtg

H.sapiens      cagtgtcccggg-----------------------------acccaccgcgtccgc------cccagcccgggtccccgc
P.troglodytes  cagtgtcccggg-----------------------------acccaccgcgtccgc------cccagcccgggtccccgc
M.mulatta      cagtgtcccggg-----------------------------acccaccgcgcccgc------cccagcccaggtccccgc
U.arctos       cgggagagggg------------------------------gccccagcc----------ctgtgccccagccccg-
B.taurus       cgggagagggg------------------------------gccccagcg----------ccgcgtcccggacccg-
S.carolinensis tggggagagggctgcaagcaagcggcagagtagtggagggcctgcaggttcgcccaccacccagccctgggatcccga
```

```
H.sapiens       gcccacccc ATGGCGAC------------------GGACGCGGCGCTACGCCGGCTTCTGAGGCTGCACCGCACGGAGAT
P.troglodytes   gcccacccc ATGGCGGC------------------GGACGCGGCGCTACGCCGGCTTCTGAGGCTGCACCGCACGGAGAT
M.mulatta       gctgaccccATGGCGGC------------------GGACGCAGCCCTGCGCCAGCTTCTGAGGCTGCACCGCACGGAGAT
U.arctos        -------cgATGGCGGGCGAGGCCCGGGCAGCTGGGGACGCGGTTCTGCGCCGCCTCCTGAGGATGTACCGCACGGAGAT
B.taurus        -------cgATGGCGGGCGAGACCCGGGCCGGTGGGGACGCGGCGCTGCGCCGCCTGCTGAGGCTGCACCGCACGGAGAT
S.carolinensis cccagccccATGGCCACCGAGACTCGGGCTGGCGGGGACTCAGCGCTGCGCCGCCTCCTGAGGCTGCACCGCACGGAGAT

H.sapiens       CGCGGTGGCCGTGGACAGCGCCTTCCCACTGCTGCACGCGCTGGCTGACCACGACGTGGTCCCCGAGGACAAGTTTCAG
P.troglodytes   CGCGATGGCCGTGGACAGCGCCTTCCCACTGCTGCACGCGCTGGCCGACCACGACGTGGTCCCCGAGGACAAGTTCCAG
M.mulatta       CGCGGTGGCCGTGGACAGCGCCTTCCCACTGCTGCACGCGCTGGCCGACCACGACGTGGTCCCCGAGGACAAGTTCCAG
U.arctos        CGCCATGGCTGTGGACAGCGCCTTCCCGCTGCTGCATGCGCTGGCTGACCATGACGTGGTCCCCGAGGACAAGTTCCAG
B.taurus        CGCGGTGGCCGTGGACAGCGCCTTCCCGCTGCTGCACGCGCTGGCCGACCACGACGTGGTCCCCGAGGAGAAGTTCCAG
S.carolinensis CGCCATGGCCGTGGACAGCGCCTTCCCGCTGCTTCACGCGCTGGTAGACCATGACGTGGTCCCTGAGGACAAGTTCCAG
```

Figure S1. Multiple sequence alignment of *AIRE* from different species. Multiple sequence alignment (*AIRE* exon 1 and the first 1kb upstream of exon 1) from six mammalian species (*Homo sapiens*, ENST00000291582.6; *Pan troglodytes*, NC_072419.1; *Macaca mulatta*, NC_041756.1; *Bos taurus*, NC_037328.1; *Ursus arctos*, NW_025929905.1; *Sciurus carolinensis*, NC_062221.1). Sequences were aligned with MAFFT using local pair option (L-INS-i). The sites of variants reported in this work and transcription factor binding sites (either predicted or confirmed for the human sequence as reported by Lovewell et al. [34] are indicated on top of the alignments.

1.2. Supplementary Tables

Table S1. Nomenclature, allele and genotype frequency of the identified *AIRE* gene promoter SNPs. Data derived from dbSNP and ENSEMBL databases.

AIRE **promoter SNPs**	**Allele frequency from the ALFA project – dbSNP database**	**Genotype frequency (count) from 1000 Genomes Project Phase 3 – ENSEMBL database**
-230Y (C/T)<br><br>rs751032	C=0.88336<br><br>T=0.11664<br><br>(sample size: 23388)	T|T: 0.026 (64)<br><br>C|C: 0.793 (1986)<br><br>C|T: 0.181 (454)
-655R (G/A)<br><br>rs117557896	G=0.99819<br><br>A=0.00181<br><br>(sample size: 18810)	NA
-261M (C/A)<br><br>rs934375604	C=0.99986<br><br>A=0.00014<br><br>(sample size: 14050)	NA
-380S (C/G)<br><br>rs371261300	C: 1.00000<br><br>G: 0.00000<br><br>(sample size: 14050)	NA
-191M (C/A)<br><br>rs1048356976	C=0.99972<br><br>A=0.00028	NA

	(sample size: 14050)	
-452Y (C/T)<br><br>rs547103905	C=0.99984<br><br>T=0.00016<br><br>(sample size: 18890)	C|C: 0.999600638977636 (2503)<br><br>C|T: 0.000399361022364217 (1)
-402S (C/G)<br><br>(not described)	NA	NA
-214M (C/A)<br><br>rs184978263	C=0.99501<br><br>A=0.00499<br><br>(sample size:14420)	C|C: 0.988 (2473)<br><br>A|C: 0.012 (31)

Table S2 . Additional clinical, genetic and immunological characteristics of the 74 APS patients.

Pt n°	Auto Abs	*AIRE* gene pattern/ protein	C1858T *PTPN22*
1	**PCA, ACA pos** TgAb,TPOAb neg	WT	neg
2	**SMA pos**<br>ANA, AMA, ARA, RAb, IAA,GADAb IA2Ab, ZnT8Ab, LKMAb, LC1Ab,<br>PCA, ACA neg	het c.607 C>T (R203X)<br>het c.834 C>G (S278R)<br>het IVS9 +6 G>A<br>het IVS9 +78 delC	neg
3	**TgAb, TPOAb pos**<br>TRGAb neg	het IVS9+78delC	neg
4	**TgAb, TPOAb,IAA pos**<br>ANA, AMA, SMA, ARA, RAb, ACA, GADAb, IA2Ab, LKMAb, LC1Ab, PCA, TRGAb,<br>ACA neg	het IVS9+6 G>A<br>het IVS9+78delC	neg
5	**ANA, TPOAb, GADA, IA2Ab pos**<br>AMA, SMA, ARA, RAb, ACA, TgAb, LKMAb, LC1Ab, PCA, TRGAb neg	het IVS9+78 delC	neg
6	**ANA, TgAb, TPOAb pos** AMA, SMA, ARA, ENA, RAb, dsDNAAb, IAA, GADAb, IA2Ab, LKMAb, LC1Ab, PCA, TRGAb neg	hom IVS9+6 G>A<br>het IVS9+78delC	neg
7	**IAA pos**<br>ANA, anti-phospholipid Ab, CeAb, β2GP1Ab, TgAb, TPOAb, GADA, TRGAb, DGPIgG neg	het<br>IVS5-31 G>A	pos
8	**TPOAb, GADAb, IA2Ab pos**<br>ANA, AMA, SMA, ANCA, ARA, RAb, TgAb, IAA, LKMAb, SLAIgG, LC1Ab, PCA, TRGAb, ACAneg	WT	pos
9	**TPOAb, PCA pos**<br>ANA, AMA, SMA, ANCA, ARA, RAb, TgAb, LKMAb, SLAIgG, LC1Ab, IFIAb, TRAb, TRGAb, ACA neg	het IVS9+78delC	neg

10	**TPOAb pos** ANA, AMA, SMA, ANCA, ARA, RAb, TgAb, IAA, GADAb, IA2Ab, LKMAb, LC1Ab, PCA, TRGAb, DGPIgG,,ACA neg	het IVS9+78delC	neg
11	**ANA, CAb, DFS70 Ab, TgAb, TPOAb pos** ASMA, ANCA, ARA, RAb, dsDNAAb, DNA, ENA, pRIBAb, phospholipid Ab, β2GP1Ab, IAA, GADAb, IA2Ab, LKMAb, LC1Ab, PCA, TRGA, ACA neg	Het IVS9+6 G>A	neg
12	**TgAb, TPOAb, TRAb pos** ANA, AMA, SMA, ANCA, ARA, RAb, LKMAb, SLAIgG, LC1Ab, PCA TRGAb, DGPIgG,ACA neg	WT	neg
13	**TPOAb pos** ANA, AMA, SMA, ANCA, ARA,RAb, dsDNAAb/DNA/ENA/pRIB Ab, TgAb, LKMAb, LC1Ab, PCA, TRAb,TRGAb,DGPIgG, ACA neg	WT	neg
14	**TgAb, TPOAb, GADA, IA2Ab pos** TRGAb neg	het IVS9+78delC	neg
15	**ANA, TPOAb, PCA, ACA pos** AMA,SMA, ARA, RAb, dsDNAAb/ENA/pRIBAb,TgAb, IAA, GADAb, IA2Ab, ZnT8Ab, LKMAb, LC1Ab, TRGAb, DGPIgG neg	WT	neg
16	NA	WT	neg
17	**TPOAb, IAA, GADAb pos** ANA, AMA, SMA, ARA, RAb, CAb, TgAb, IA2Ab, LKMAb, LC1Ab, APCA, TRGAb, ACA neg	het IVS9+6 G>A<br>hom IVS9+78delC	neg
18	TgAb, TPOAb, TRGAb neg	WT	pos
19	**TgAb, TPOAb, IAA, GADAb, IFIgG, PCA pos** ANA, AMA, SMA, ARA, RAb, CeAb, IA2Ab, LKMAb, LC1Ab,TRGAb, DGPIgGACA neg	het IVS9+78 delC	pos
20	**ANA, TgAb, TPOAb, GADA, IA2Ab, APCA pos** AMA, ARA, SMA, RAb, CeAb, LKMAb, LC1Ab, IFIAb, TRGAb, ACA DGPIgG, neg	WT	neg
21	**ANA, TgAb, TPOAb, IAA, GADAb, IA2Ab pos** AMA, SMA, ARA, RAb, dsDNAAb, CAb, LKMAb, LC1Ab, PCA, TRGAb, ACA neg	het IVS9+78delC	pos
22	**TPOAb,IAA, GADAb pos** ANA,AMA, SMA,ARA, RAb,,CeAb, TgAb,IA2Ab, LKMAb,LC1Ab, PCA,TRGAb, DGPIgG, ACA, 21-OHAb neg	het c.834 C>G (S278R)<br>het IVS9+78delC	neg
23	**TPOAb,IAA, IA2Ab pos** ANA,AMA, SMA, ARA, RAb,,CeAb, TgAb,GADAb, LKMAb,LC1Ab, PCA,TRGAb, ACA neg	het c.834 C>G (S278R)	neg
24	**IAA,GADAb, IA2Ab, IFIAb,**	WT	neg

	**PCA pos**<br>ANA,AMA,SMA,<br>ARA,RAb,CAb,<br>TgAb, TPOAb,<br>LKMAb, LC1Ab,<br>TRAb, TRGAb,<br>DGPIgG, ACA, 21-OHAb neg		
25	NA	het IVS9+78delC	pos
26	ANA, SMA,<br>ANCA, RAb, LKMAb,SLAIgG, LC1Ab neg	WT	neg
27	**IAA, IA2Ab pos**<br>ANA,AMA,SMA,<br>ANCA,ARA,RAb<br>CeAb,,TgAb,,<br>TPOab,GADAb,LKMAb, LC1Ab, PCA, TRGAb,<br>ACA,21-OHAb neg	hom IVS9+78delC	neg
28	**IAA, GADAb,**<br>**IA2Ab pos**<br>ANA,AMA,SMA,<br>ARA, RAb,CeAb, β2GP1Ab,TgAb,<br>TPOAb,<br>LKMAb ,LC1Ab,<br>PCA,TRGAb,<br>DGPIgG,<br>21-OHAb neg	het IVS9+6 G>A<br>het IVS9+78 delC	neg
29	**TPOAb,IAA pos**<br>ANA, AMA, SMA, ARA<br>RAb, CeAb,<br>TgAb,GADAb,<br>IA2Ab,LKMAb,<br>LC1AbPCA,<br>TRAb,TRGAb,<br>ACA,21-OHAb neg	WT	neg
30	**TgAb,TPOAb,**<br>**IAA pos**<br>GADAb,IA2Ab,<br>TRGAb,<br>21-OHAb neg	het IVS9+78delC	neg
31	**TPOAb,**<br>**LKMAb pos**<br>ANA,AMA,<br>SMA, ANCA,<br>ARA,RAb,<br>TgAb,SLAIgG<br>LC1Ab,PCA,<br>TRGAb,<br>DGPIgG neg	het IVS9+78delC	neg
32	**ANA,TgAb,**<br>**TPOAb,**<br>**ACA pos**<br>AMA,SMA,<br>ANCA,ARA,<br>RAb,IAA,<br>GADAb,IA2Ab,<br>LKMAb,LC1Ab,<br>IFIAb,PCA,<br>TRAb,TRGAb,	WT	neg

	DGPIgG neg		
33	**TPOAb,IAA, IA2Ab, TRGAb pos** ANA,AMA, SMA,CeAb, TgAb,GADAb, PCA,TRAb, DGPIgG,ACA 21-OHAb neg	het IVS9+78delC	neg
34	**IAA, IA2Ab pos** ANA,AMA, SMA,ARA, RAb, CeAb,TgAb TPOAbGADAb LKMAb, PCA, LC1Ab, TRGAb DGPIgGACAneg	het IVS9+78delC	pos
35	NA	het IVS9+6 G>A	pos
36	NA	WT	neg
37	**TPO, IAA pos** TgAb, GADAb, IA2Ab, TRGAb, 21-OHAb neg	het IVS9+78delC	neg
38	**TRGAb pos** TgAb, TPOAb neg	het IVS9+6 G>A het IVS9+78delC	neg
39	**IAA,GADAb pos** ANA,AMA, SMA,ARA,RAb, CeAb,TgAb, TPOAb,IA2Ab LKMAb, LC1Ab, PCA,TRGAb, DGPIgG ACA neg	WT	neg
40	**ANA,TPOAb, GADAb,IA2Ab pos** AMA,SMA, ARA,RAb, dsDNAAb, CeAb,TgAb, LKMAb,LC1Ab, PCA,TRGAb DGPIgG, ACA neg	het IVS9+78delC	neg
41	**TgAb, TPOAb, IAA pos** ANA, AMA, SMA, CAb, GADA, IA2Ab, APCA, TRGAb,ACAneg	het IVS9+6 G>A hom IVS9+78delC	neg
42	**TgAb, TPO Ab, IAA, GADAb, IA2Ab pos** ANA, AMA, SMA, ARA, DGP-IgGAb, CeAb, LKMAb, LC1Ab, PCA, TRGAb, ACAneg	het c.834 C>G (S278R) het IVS9+6 G>A het IVS9+78delC	pos
43	**IAA, GADAb pos** TgAb, TPOAb, IA2Ab, TRGAb, ACA neg	hom IVS9+78delC	neg
44	**ANA, SMA, TgAb, GADAb, IA2Ab, pos** AMA, CeAb, TPOAb, APCA, TRGAb, ACA 21-OHAb neg	het IVS9+78delC	neg
45	**IAA, GADAb pos** ANA, AMA, SMA, ARA, RAb, CeAb, TgAb, TPOAb, IA2Ab, LKMAb, LC1Ab, PCA, TRGAb, DGPIgG,	WT	pos

	21-OHAb neg		
46	**TPOAb,PCA pos**<br>ANA, AMA, ASMA, ARA, RAb, CAb, TgAb, GADAb, IA2Ab, LKMAb, LC1Ab, IFIgG, TRAb, TRGAb,ACA neg	het c.834 C>G (S278R)<br>het IVS9+6 G>A<br>het IVS9+78delC	neg
47	**ANA, CeAb, TgAb, TPOAb, GADAb, IA2Ab, PCA pos**<br>AMA, SMA, ARA,RAb, β2GP1Ab, LKMAb, LC1Ab, IFIAb, TRGAb. ACA neg	hom IVS9+78delC	neg
48	**IA2Ab, ACA pos** TgAb, TPOAb, IAA, GADAb, ZnT8Ab, TRGAb neg	het c.834 C>G (S278R)	neg
49	**TgAb, TPOAb, IAA GADAb, PCA pos**<br>ANA, AMA,<br>SMA, ARA, RAb, CeAb, IA2Ab, LKMAb, LC1Ab, IFIAb, TRGAb, ACA neg	het IVS9+78 delC	neg
50	**GADAb pos**<br>TgAb, TPOAb, IAA, IA2Ab, TRGAb neg	het IVS9+78delC	pos
51	**TgAb, TPOAb, IFIAb, APCA pos;** ANA, AMA, SMA, ARA, RAb, CeAb, IAA, GADAb, IA2Ab, LKMAb, LC1Ab, TRGAb,ACA,<br>DGPIgG, neg	het c.834 C>G (S278R)	neg
52	**ANA, TgAb, TPOAb, IAA, GADAb pos**<br>AMA, SMA,<br>ARA, RAb, CeAb, IA2Ab, LKMAb, LC1Ab, APCA, TRAb, TRGAb, ACA,<br>21-OHAb neg	het IVS9+6 G>A<br>hom IVS9+78delC	neg
53	**TgAb, TPOAb pos**<br>ANA, AMA, ANCA, ENA, EMA, dsDNAAb, PCA, TRGAb, DGPIgG neg	hom IVS5+14 C>T	neg
54	NA	hom IVS5+14 C>T<br>het IVS9+6 G>A	neg
55	**TgAb pos** ANA, ANCA, DNA, ENA, RAb, TPOAb, IAA, GADAb, IA2Ab, ZnT8Ab, TRAb, TRGAb neg	het c.834 C>G<br>(S278R) het<br>IVS13-55 A>G<br>het IVS14-127 G>C	neg
56	**ACA pos**<br>ANA, AMA, SMA, ANCA, ARA, RAb, DNA/ENA/pRIB Ab, TgAb, TPOAb, IAA, GADAb,,IA2Ab, ZnT8Ab, LKMAb, IFIAb, PCA, TRGAb, CCPAb neg	het IVS9+78delC<br>het c.1333 C>T<br>(R445W)	neg
57	**TgAb, TPOAb pos**<br>ANA, AMA, SMA, ARA, ENA, Rab, dsDNAAb, SCL-70Ab, ICA, IA2Ab, LKMAb, PCA, TRGAb, ACA neg	het IVS9+6 G>A<br>hom IVS9+78delC	neg
58	**EMA, CeAb, TgAb, TPOAb, IAA, GADAb, IA2Ab, pos**<br>ANA, ANCA, dsDNAAb, DNA, ENA, pRIB Ab, phospholipidAb, β2GP1Ab,TRGb, DGPIgG neg	het IVS9+78 delC	neg
59	NA	het c.834 C>G<br>(S278R)<br>het IVS9+6 G>A<br>het IVS9+78 delC	pos
60	ANA, TgAb, TPOAb, IAA, GADAb, IA2Ab, ZnT8Ab, ACAneg	het IVS9+78delC	pos
61	**DGPIgG, adrenal Ab pos**<br>ANA, AMA, SMA, ANCA, ARA, ENA, RAb, anti-phospholipid Ab, CeAb, β2GP1Ab,TgAb, TPOAb, IAA, IA2Ab, GADAb, ZnT8Ab,LKMAb, LC1Ab, PCA, TRGAb neg	het c.834 C>G (S278R)<br>het IVS14 -127 G>A	neg

62	NA	het c.834 C>G (S278R)<br>het c.892 G>A<br>(Z298K)	neg
63	NA	WT	neg
64	**TgAb, TPOAb, 21-OHAb pos** TRGAb, ACA neg	het IVS9 +6 G>A	pos
65	**IA2Ab pos**<br>TgAb, TPOAb, GADAb TRGAb ACA, 21-OH Ab neg	het IVS9 +78 delC	neg
66	NA	het IVS9 +78 delC	neg
67	NA	WT	pos
68	**TPOAb, TRGAb pos**, TgAb, GADA, IA2Ab, DGPIgG, ACA, 21-OHAb neg	het IVS9 +78 delC	neg
69	NA	WT	neg
70	**TgAb, TPOAb, IAA pos** IA2Ab, GADAb, TRAb, TRGAb,ACA neg	het c.834 C>G<br>(S278R)<br>het IVS9 +6 G>A<br>het IVS9 +78 delC	neg
71	**TgAb, TPOAb pos**<br>IAA, GADAb IA2Ab, TRAb, TRGAb neg	het c.834 C>G<br>(S278R)<br>hom IVS9+78delC	neg
72	**ASMA, TgAb, TPOAb, IAA pos**<br>ANA, AMA, ARA RAb, CeAb, GADAb, IA2Ab, LKMAb, LC1Ab, IFIAb, PCA, TRGAb, ACA, 21-OHAb neg	het IVS9+78delC	neg
73	**IAA GADAb IA2Ab pos**<br>ANA, AMA, SMA, ARA, RAb, CeAb, TgAb, TPOAb, LKMAb, LC1Ab, PCA, TRGAb,DGPIgG, ACA neg	het IVS9+78delC	pos
74	NA	het IVS5 +14 C>T<br>het c.834 C>G<br>(S278R)<br>het IVS13 -55 A>G	neg

WT, wild type; neg, negative; pos, positive; NA, not available.

Table S3. Comparison of SNPs between patients and healthy controls by statistical analysis.

```
. tab groups _230Y, col chi exact

+-------------------+
| Key               |
|-------------------|
|     frequency     |
| column percentage |
+-------------------+

           |         -230Y
    groups |        no        yes |     Total
-----------+----------------------+----------
   control |        62         19 |        81 
           |     53.45      48.72 |     52.26 
-----------+----------------------+----------
   patient |        54         20 |        74 
           |     46.55      51.28 |     47.74 
-----------+----------------------+----------
```

```
     Total |       116         39 |       155 
           |    100.00     100.00 |    100.00 

          Pearson chi2(1) =   0.2618   Pr = 0.609
           Fisher's exact =                 0.711
   1-sided Fisher's exact =                 0.372

. tab groups _230T , col chi exact

+-------------------+
| Key               |
|-------------------|
|     frequency     |
| column percentage |
+-------------------+

           |         -230T
    groups |        no        yes |     Total
-----------+----------------------+----------
   control |        78          3 |        81 
           |     52.00      60.00 |     52.26 
-----------+----------------------+----------
   patient |        72          2 |        74 
           |     48.00      40.00 |     47.74 
-----------+----------------------+----------
     Total |       150          5 |       155 
           |    100.00     100.00 |    100.00 

          Pearson chi2(1) =   0.1241   Pr = 0.725
           Fisher's exact =                 1.000
   1-sided Fisher's exact =                 0.543

. tab groups _655R , col chi exact

+-------------------+
| Key               |
|-------------------|
|     frequency     |
| column percentage |
+-------------------+

           |         -655R
    groups |        no        yes |     Total
-----------+----------------------+----------
   control |        73          8 |        81 
           |     50.00      88.89 |     52.26 
-----------+----------------------+----------
   patient |        73          1 |        74 
           |     50.00      11.11 |     47.74 
-----------+----------------------+----------
     Total |       146          9 |       155 
           |    100.00     100.00 |    100.00 

          Pearson chi2(1) =   5.1388   Pr = 0.023
           Fisher's exact =                 0.035
   1-sided Fisher's exact =                 0.024
```

The frequence of -655R SNP is significantly different between controls and patients: 89% vs 11.0%, respectively (p=0.024).

```
. tab groups _655A , col chi exact

+-------------------+
| Key               |
|-------------------|
|     frequency     |
| column percentage |
+-------------------+

           |   -655A
    groups |        no |     Total
-----------+-----------+----------
   control |        81 |        81
           |     52.26 |     52.26
-----------+-----------+----------
   patient |        74 |        74
           |     47.74 |     47.74
-----------+-----------+----------
     Total |       155 |       155
           |    100.00 |    100.00


. tab groups _261M , col chi exact

+-------------------+
| Key               |
|-------------------|
|     frequency     |
| column percentage |
+-------------------+

           |         -261M
    groups |        no        yes |     Total
-----------+----------------------+----------
   control |        81          0 |        81
           |     52.60       0.00 |     52.26
-----------+----------------------+----------
   patient |        73          1 |        74
           |     47.40     100.00 |     47.74
-----------+----------------------+----------
     Total |       154          1 |       155
           |    100.00     100.00 |    100.00

          Pearson chi2(1) =   1.1017   Pr = 0.294
           Fisher's exact =                 0.477
   1-sided Fisher's exact =                 0.477

. tab groups _380S , col chi exact

+-------------------+
| Key               |
|-------------------|
|     frequency     |
| column percentage |
+-------------------+
```

```
           |         -380S
    groups |        no        yes |     Total
-----------+----------------------+----------
   control |        81          0 |        81
           |     52.60       0.00 |     52.26
-----------+----------------------+----------
   patient |        73          1 |        74
           |     47.40     100.00 |     47.74
-----------+----------------------+----------
     Total |       154          1 |       155
           |    100.00     100.00 |    100.00

          Pearson chi2(1) =   1.1017   Pr = 0.294
           Fisher's exact =                 0.477
   1-sided Fisher's exact =                 0.477

. tab groups _191M , col chi exact

+-------------------+
| Key               |
|-------------------|
|     frequency     |
| column percentage |
+-------------------+

           |         -191M
    groups |        no        yes |     Total
-----------+----------------------+----------
   control |        81          0 |        81
           |     52.60       0.00 |     52.26
-----------+----------------------+----------
   patient |        73          1 |        74
           |     47.40     100.00 |     47.74
-----------+----------------------+----------
     Total |       154          1 |       155
           |    100.00     100.00 |    100.00

          Pearson chi2(1) =   1.1017   Pr = 0.294
           Fisher's exact =                 0.477
   1-sided Fisher's exact =                 0.477

. tab groups _402S , col chi exact

+-------------------+
| Key               |
|-------------------|
|     frequency     |
| column percentage |
+-------------------+

           |         -402S
    groups |        no        yes |     Total
-----------+----------------------+----------
   control |        81          0 |        81
           |     52.60       0.00 |     52.26
-----------+----------------------+----------
   patient |        73          1 |        74
```

```
           |     47.40     100.00 |     47.74 
-----------+----------------------+----------
     Total |       154          1 |       155 
           |    100.00     100.00 |    100.00 

          Pearson chi2(1) =   1.1017   Pr = 0.294
           Fisher's exact =                 0.477
   1-sided Fisher's exact =                 0.477

tab groups tot variations, col chi exact

+-------------------+
| Key               |
|-------------------|
|     frequency     |
| column percentage |
+-------------------+

           |       tot* variations
    groups |         0          1 |     Total
-----------+----------------------+----------
   control |        51         30 |        81 
           |     50.50      55.56 |     52.26 
-----------+----------------------+----------
   patient |        50         24 |        74 
           |     49.50      44.44 |     47.74 
-----------+----------------------+----------
     Total |       101         54 |       155 
           |    100.00     100.00 |    100.00 

          Pearson chi2(1) =   0.3612   Pr = 0.548
           Fisher's exact =                 0.614
   1-sided Fisher's exact =                 0.333
```

*refers to the number of individuals carrying at least one *AIRE* promoter SNPs
